# Adults, but not preschoolers or toddlers integrate situational constraints in their action anticipations: a developmental study on the flexibility of anticipatory gaze

**DOI:** 10.1007/s10339-021-01015-8

**Published:** 2021-03-24

**Authors:** Kerstin Ganglmayer, Marleen Haupt, Kathrin Finke, Markus Paulus

**Affiliations:** 1 Department Psychology, Developmental Psychology, Ludwig Maximilians-Universität München, Leopoldstr. 13, 80802 Munich, Germany; 2 Department Psychology, General and Experimental Psychology, Ludwig-Maximilians-Universtität München, Munich, Germany; 3 Hans-Berger Department of Neurology, University Hospital Jena, Jena, Germany

**Keywords:** Action anticipation, Situational constraints, Eye-tracking, Predictive coding

## Abstract

Recent theories stress the role of situational information in understanding others’ behaviour. For example, the predictive coding framework assumes that people take contextual information into account when anticipating other’s actions. Likewise, the teleological stance theory assumes an early developing ability to consider situational constraints in action prediction. The current study investigates, over a wide age range, whether humans flexibly integrate situational constraints in their action anticipations. By means of an eye-tracking experiment, 2-year-olds, 5-year-olds, younger and older adults (together *N* = 181) observed an agent repeatedly taking one of two paths to reach a goal. Then, this path became blocked, and for test trials only the other path was passable. Results demonstrated that in test trials younger and older adults anticipated that the agent would take the continuous path, indicating that they took the situational constraints into account. In contrast, 2- and 5-year-olds anticipated that the agent would take the blocked path, indicating that they still relied on the agent’s previous observed behaviour and—contrary to claims by the teleological stance theory—did not take the situational constraints into account. The results highlight developmental changes in human’s ability to include situational constraints in their visual anticipations. Overall, the study contributes to theories on predictive coding and the development of action understanding.

We constantly allocate our attention predictively, either when performing our own actions or when observing actions of others (Flanagan and Johannson 2003). The ability to anticipate others’ behaviour—that is, to proactively attend to a future state of an action—has been proposed to be a central aspect of human social cognition. For example, it has been suggested that the ability to anticipate others’ actions enables efficient interaction and is thus an essential capacity for everyday social functioning (Bekkering et al. 2009; Sebanz and Knoblich 2009). Consequently, psychological research aims at understanding the developmental basis and psychological mechanisms that support action anticipation (e.g. Ambrosini et al. 2011; Ambrosini et al. 2015; Eshuis et al. 2009; Paulus 2012; Ruffman et al. 2012).

In this paper, the ability to include situational constraints into action anticipations is investigated across a wide age span. Situational constraints reduce our action possibilities and make certain actions more likely than others (Van Overwalle 2010). Taking into account situational constraints is thus central for action understanding. For example, in case the usual way to the supermarket is blocked due to a construction site, we are able to predict that people will take the alternative route. We live in a social world that is constantly confronting us with changing environmental conditions that determine our possible actions. Information provided by situational constraints is therefore especially informative when processing another’s action. An important developmental task is therefore to take contextual changes into account when predicting others’ behaviour. Yet, there is limited evidence on how changes in situational constraints affect action anticipation, and how this changes in the course of development.

The ability to integrate situational constraints into action anticipations can be nicely framed within a predictive coding perspective. According to predictive coding theories, information provided by the context is especially informative for action anticipation (Clark 2013a; Kilner et al. 2007). Predictive coding theories claim that a bidirectional hierarchically structured system in the cognitive system constantly compares bottom-up sensory input with top-down predictions (Clark 2013a). Top-down predictions rely on higher-level knowledge, i.e. our concept of what the world “typically” looks like (in short “priors”; Friston 2010). The amount of sensory input that is not predicted by our priors is reported backwards as a prediction error, in order to improve future top-down predictions. Thereby it has been claimed that when anticipating other’s actions, context information functions as a very reliable prior (Kilner et al. 2007). The perception of contextual information is especially fast (Oliva 2005). The perceptual and conceptual meaning of a scene, also referred to as the *gist* of a scene, can be recognized within 100 ms (Oliva 2005). This implies that in most cases, context information is already available before we observe someone’s action. That is, a set of context-informed priors is already active and “ready” to predict and thus influences our perception through top-down predictions (Clark 2013a). In sum, context information is supposed to be highly informative and to constitute an important factor in the processing of others’ actions. Thereby context information can include information about the background or the setting of a scene (e.g. Wurm and Schubotz 2012), but also information about situational constraints, such as an obstacle that might block another’s way.

Prior research demonstrated that infants and young children take contextual information into account when anticipating other’s actions. For example, it has been shown that infants adapt their anticipations to the type of agent (Kanakogi and Itakura 2011) or they differentiate between the type of hand grip (precision or whole hand grip) when anticipating which of two objects a hand is going to grasp (Ambrosini et al. 2013). Although there is ample research on the influence of contextual information on action anticipation in infants (e.g. Adam et al. 2016, 2017; Ambrosini et al. 2013; Gampe and Daum 2014; Gredebäck et al. 2009; Henrichs et al. 2014), children (e.g. Ganglmayer et al. 2019a) and adults (e.g. Ambrosini et al. 2015; Eshuis et al. 2009), it is an open question how and whether situational constraints are processed for action anticipations. One recent study suggested that adults consider situational constraints in verbal action prediction (Stapel et al. 2012). Adults were better in verbally stating how another’s action would continue, when the action was constrained by the situational context (Stapel et al. 2012). However, recent work indicates differences between time-consuming verbal reasoning and action anticipation (e.g. Apperly and Butterfill 2009; Schuwerk and Paulus 2016) so that a direct assessment of visual action anticipations is required.

It has further been discussed whether infants have an inborn expectation that others act efficiently (Gergely and Csibra 2003; Ruffman 2014). If this were true, infants should process situational constraints when anticipating others’ actions from early on. Although looking time studies are in line with this proposal (e.g. Csibra et al. 1999; Gergely et al. 1995; Skerry et al. 2013; for related work, see also Liu et al. 2017), it is unclear whether young children indeed consider situational constraints in their action anticipations. While some evidence supports this claim (e.g. Biro 2013), other studies have found no evidence for it (e.g. Paulus et al. 2011) and provided alternative explanations (Ruffman 2014; van Overwalle 2010). For example, Paulus et al. (2011) presented 9-month-olds and adults with a cow repeatedly taking the longer of two possible paths to reach a goal, as the shorter path was impassable. However, when the context changed and both paths were passable, in the first trial infants and adults still anticipated that the cow would continue taking the longer path, although the shorter one would have been more efficient. This suggests that both adults and infants did not immediately take the change of the situational constraints into account when anticipating the agent’s behaviour. However, for both studies (Biro 2013; Paulus et al. 2011), situational constraints were only present in learning trials, whereas in the critical test trials, the situational constraints were gone and it was assessed whether this led to a change in anticipatory behaviour within participants. Direct empirical evidence of whether young children and adults adapt their anticipations to suddenly occurred situational restrictions is still missing.

Moreover, it is unclear whether and to what extent older adults integrate situational constraints into their action anticipations. From a predictive coding point of view, our top-down predictions should improve through lifelong experience (Clark 2013a). Prediction errors, i.e. the amount of sensory input that was not explained by higher-level predictions, shape our future predictions and are consequently important for learning. Thus, with increasing age our predictive system should become more accurate and reliable. We should become better in weighing the predictive power of different information sources, which is also called “hyperpriors” (Ambrosini et al. 2015; Clark 2013a). Hyperpriors are priors on a higher level of abstraction and include “general knowledge” of the world (e.g. Clark 2013a; Hohwy et al. 2008); for example, the higher-level knowledge that people take an alternative route to get to the supermarket, when their usual way is blocked because of a construction site. Hyperpriors must be built and learned through lifelong experience (Clark 2013a). This indicates that older adults might become better in their action anticipation abilities, due to their lifelong experience. Interestingly, an age-related increase in action anticipation abilities across the lifespan has indeed been observed by Wermelinger et al. (2019). Older participants were better in anticipating an unfamiliar action. However, others suggested a decline of action prediction abilities at older ages (Diersch et al. 2012, 2016). Nevertheless, in neither of the mentioned studies did participants have to take the occurrence of situational constraints into account, leaving the question open of how this ability might develop in older ages. Such a decrease reported in the latter studies is also in line with age-related declines in inhibition and processing speed (see, for example, Gazzaley et al. 2008; Zelazo et al. 2004). Tasks that include changes in the environment, such as the occurrence of situational constraints, require fast and flexible adaptation, implying that an age-related decline in action anticipation abilities might be related to other age-related factors.

Given such opposing results, it is not clear how well older adults can integrate occurred situational constraints in their action anticipations. Based on the predictive coding theory, it could be assumed that the ability to flexibly integrate situational constraints in predictions linearly increases with age, based on the continuously built-up experience.

However, at older ages, a decline in executive capabilities might increasingly counteract such increase. Taken together, the ability to flexibly integrate situational constraints when anticipating others’ actions might change across the lifespan. It might linearly increase with increasing lifelong experience, or it might take the form of an inverted u-shaped trajectory due to an age-related decline in cognitive functions (see also Zelazo et al. 2004). Thus, an investigation across the lifespan is necessary in order to empirically test the predictions made by theories on predictive coding and action processing.

## The current study

The current study addresses the question of whether participants at different age levels take situational constraints into account when anticipating another’s action. Do people flexibly adapt their visual anticipations to suddenly occurring situational constraints? And how does this ability develop across a wide age span? Two-year-olds, 5-year-olds, younger and older adults were included in the current eye-tracking study. In order to investigate action processing, we relied on a paradigm that allows assessing action anticipations already in younger children (Daum et al. 2012; Paulus et al. 2011). More specifically, the paradigm by Paulus et al. (2011) was used and adapted for the study’s purpose. However, instead of presenting participants with an animated agent taking the longer of two paths (as the shorter was impassable), participants repeatedly observed an animated agent taking one of two equally long and passable paths to reach a goal. Importantly, the agent always took the same of the two paths. After several repetitions, this path was blocked and thus impassable. Hence, the agent had to take the other path to reach its goal. We measured whether participants visually anticipated that the agent would take the other path, as the former was impassable.

We decided to include 2-year-old children, as developmental theories would make different predictions of their performance. This is relevant for developmental theorizing as some theories would assume that they consider situational constraints (Gergely and Csibra 2003), whereas others would suggest differently (Ruffman 2014).

We further included 5-year-olds as from a predictive coding perspective older children should improve their anticipation abilities as they have more lifelong experience in observing others actions. Also previous studies suggest that from around 3 years of age, children become more sophisticated in their action anticipation abilities. For example, 3.5-year-olds integrate verbal information in their action anticipations (Paulus et al. 2017). Similarly, Daum et al. (2012) observed that from 3 years of age children anticipate the specific goal of an action even though it changed location, by using a similar paradigm as in the current study. We expect 5-year-olds to integrate the situational constraints in their anticipations and perform better than the 2-year-old toddlers, but might not perform as well as adults.

To investigate action anticipation abilities across a wide age span in order to get a first indicator about developmental changes over the course of life, younger and older adults were also included. Predictive coding theory would assume that lifelong experience is beneficial for predictions and that action anticipation abilities thus improve throughout the lifespan (Clark 2013a). If this is true, we would predict a rather linear improvement of action anticipation ability from early childhood into later adulthood. However, if the flexible integration of situational constraints into action anticipations is related to an age-related decline in cognitive factors, one would expect a decrease in action anticipation abilities compared to younger adults, leading to an inverted u-shaped trajectory over the four age groups (Gazzaley et al. 2008; Zelazo et al. 2004).

## Method

The preprocessed eye-gaze data of the study is available at https://osf.io/dpemf/?view_only=dc366915ba724c29922960eea3e2a96f. Demographic information is not included in the data set, due to protection of data privacy and to prevent inferences on individual data.

### Participants

The final sample comprised 181 participants. It consisted of 42 2-year-olds (mean age = 24.33 months, *SD* = 0.72, range = 23–26 months), 47 5-year-olds (mean age = 60.87 months, *SD* = 1.26, range = 58–66 months), 45 younger adults (mean age = 25.91 years, *SD* = 6.81, range = 18–45 years) and 47 older adults (mean age = 71.51 years, *SD* = 4.51, range = 61–78 years). Additionally, seven 2-year-olds, two 5-year-olds, five younger and three older adults were excluded. Reasons for exclusions were fussiness among the children samples (*n* = 4), problems with eye-tracking or insufficient data (*n* = 4), and experimenter error (*n* = 9). Prior to data acquisition, the sample size was determined based on a power analysis with G*Power 3.1.9.2 (Faul et al. 2009) with *α* = 0.05 and power = 0.80. For the one-way between-subjects’ ANOVA with a medium effect size of *f* = 0.25 and four groups, a sample size of 180 participants was estimated.

Informed written consent was given by participants or their caregivers prior to testing. The study was approved by the local ethics board. Adults and children were recruited via birth records, public announcements and from participant pools. Travel costs were reimbursed for children and older adults; student participants obtained monetary compensation or course credit. Children also received a small present. All participants came from or around a larger city in Europe.

To control for a possible age-related cognitive impairment indicating first signs of pathological neurodegeneration, the Mini-Mental State Examination (MMSE; Folstein et al. 1975; maximum score of 30 and a cut-off criterion of score ≥ 24 following Kochhann et al. 2010) was applied in the older adults.

### Stimuli

Stimulus material consisted of two introductory movies, five learning movies, a “blocking” movie (in which one of the paths gets interrupted) and three test movies. The movies were created with Adobe Animate CC and Adobe Illustrator (Adobe Systems Inc., San Jose, CA).

**Fig.1:**
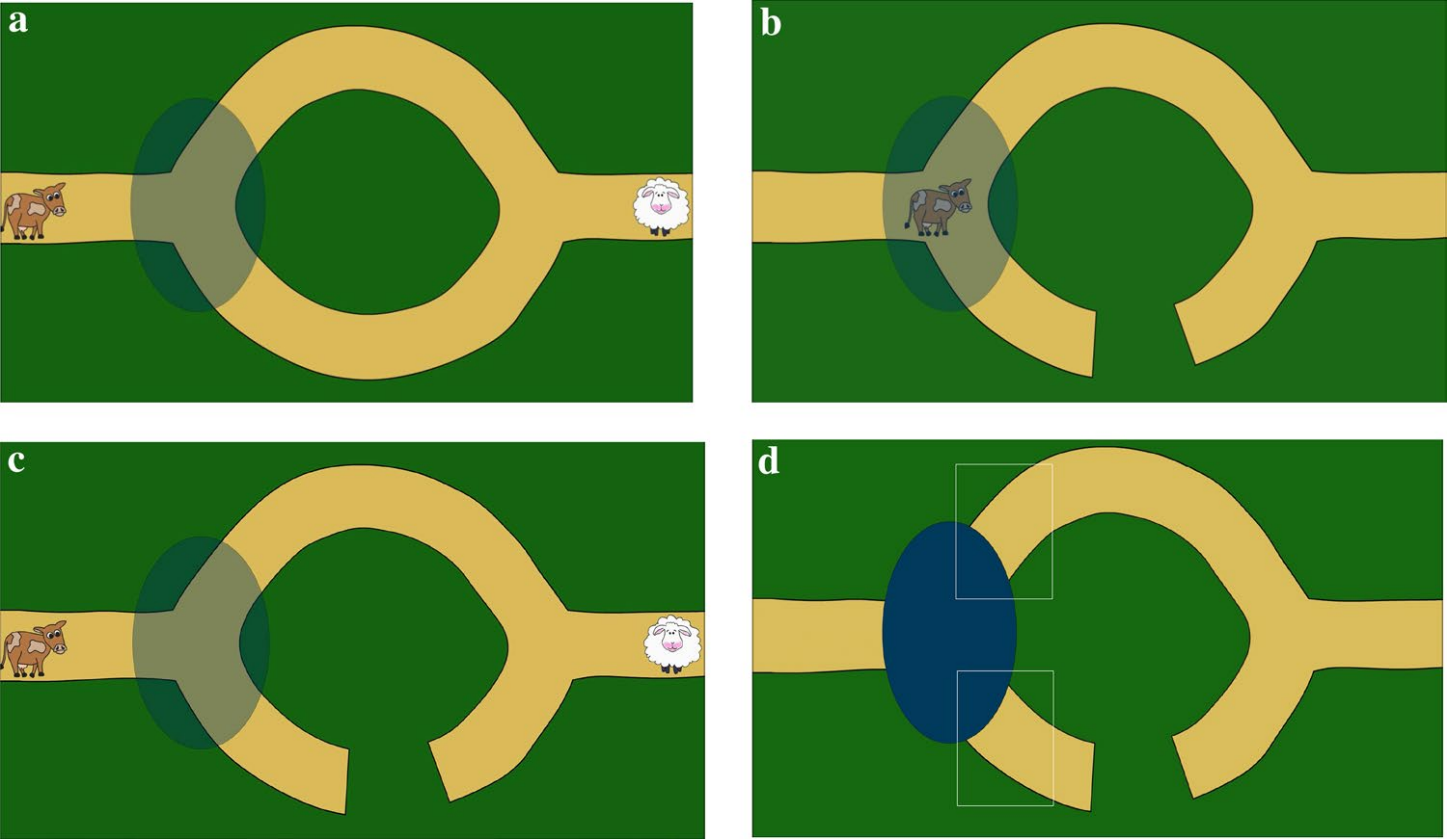
**a** Example of a learning movie: the agent (cow) is situated on the left, the goal (sheep) on the right-end side of the path. The transparent occluder (which turns opaque before the cow starts to walk) overlies the crossroad of the two paths. **b** Example of a “blocking” movie: the lower path has already become interrupted and the cow has reached the crossroad (with the occluder being transparent). **c** Example of the beginning of a test movie. **d** Illustration of the areas of interest for analysis in the test trials: white boxes indicate areas of interest

Two introductory movies familiarized participants with the set-up and presented participants with two equally long paths, leading from the left to the right side of the screen. Both paths merged into one single path at their beginnings and ends (see Fig. 1). An occluder overlaid the crossroad on the left side. The occluder was employed to elicit anticipatory eye movements and to avoid constant fixation on the agent (see Paulus et al. 2011; von Hofsten et al. 2007). At the beginning of the movie, the occluder was transparent and a cow was situated on the far left side of the path. Then, the cow jumped up two times. Immediately the transparent occluder turned opaque and the cow started to walk towards the occluder, disappeared behind it for 1.3 s, and reappeared on one of the two paths. It then walked towards the far right side and finally left the screen. This sequence lasted for 14 s. The video was presented two times, with the cow taking one of the two paths in the first and the other path in the second video. This provided participants with the information that the cow can walk on both paths and does not walk on the green surface. The order of which of the paths the cow took first was counterbalanced in randomized order between participants.

The movies of the learning phase contained the same set-up as the introductory movie, but additionally a sheep was situated at the far right side of the single path (see Fig. 1a). At the beginning, the cow jumped up twice and a voice stated “Oh a sheep, I want to get to the sheep”. Then, the sheep wiggled and moved along the path off the screen at the right side. Shortly afterwards, the occluder turned opaque and the cow started to walk towards the sheep, taking one of the two paths. Which path the cow took (upper or lower path) was counterbalanced between participants. The video of the learning trial was presented five times in a row with the cow always taking the same of the two paths.

In the following “blocking” movie, the path that was taken by the cow in the learning trials became interrupted. Participants observed the two paths, including the transparent occluder and the cow situated at the far left side of the path. Additionally, a rattle sound played for 3 s to attract participants’ attention. Afterwards, a piece of the path vanished (see Fig. 1b). This was accompanied by a triangle sound to draw participants’ attention towards the newly appeared gap in the path. To provide participants with the information that the cow was also aware of the newly appeared gap, the voice stated, “Oh what happened there?” after 3 s. Then, the cow started to move towards the crossroad with the occluder staying transparent. When it reached the crossroad it stopped, looked at the interrupted path and the voice stated, “Ah now it’s not passable anymore”. This was done in order to make participants aware that the cow cannot walk across the gap. The verbal cues were included to make the plot clearer and to emphasize the occurred situational constraint. This scene was shown for another 4 s. In total, the movie lasted 19.5 s.

The test movies started exactly like the movies of the learning trials, except that the familiarized path was now interrupted (see Fig. 1c). The cow jumped up two times and stated again “Oh a sheep, I want to get to the sheep”, the occluder turned opaque and the cow started to move towards the occluder, disappeared and did not reappear throughout the rest of the movie (4.5 s). The fact that the cow did not reappear from the occluder in the test trials ensured that participants did not learn about any alternative behaviour of the cow. One whole test movie lasted a total of 14 s.

The stimuli were additionally piloted within a sample (*n* = 14) of 3- to 6-year-olds (mean age = 4.29) to assess whether children “understand” the stimuli correctly. Children observed the cow once taking the upper path and once taking the lower path to reach the goal. Afterwards, the blocking movie was presented, showing that one of the paths becomes blocked (see description of the “blocking” movie above). Subsequently, one test movie was shown (with the corresponding path blocked). After the cow’s disappearance behind the occluder during the test trial, children were explicitly asked “Which path can the cow take now?”. Almost all of the children (*n* = 13; 93%) gave correct answers by pointing towards the continuous path or verbally stating so, with χ^2^(1) = 10.29, *p* = 0.001. Therefore, we assume that (at least by the preschool years) children understood the whole set-up as well as that the cow could not walk along the interrupted path.

### Setting and Procedure

Two-year-olds were either seated on an age-appropriate car seat (which was attached to a regular chair) or on their parent’s lap in case they did not want to sit alone. Five-year-olds, younger and older adults were seated on a regular chair in front of the monitor. For all participants, the distance to the monitor was 60–65 cm. For recording participants’ eye movements, a corneal reflection eye-tracker (Tobii Pro TX 300, Tobii Technology, Sweden) was used. It recorded eye-gaze data at 120 Hz with an average accuracy of 0.4° visual angle. The software Tobii Studio (Tobii Technology, Sweden) was used for video presentation. For calibration procedure, 2-year-olds received a 5-point calibration (due to attention reasons) and 5-year-olds, younger and older adults a 9-point calibration. Three of the 5-year-olds received a 5-point calibration due to experimenter error. After that, all age groups were told that they were going to watch a short movie and that they should watch that movie attentively.

For familiarization with the set-up and for providing participants with the information that the cow was able to take both possible paths, the two introductory movies were shown first. Afterwards five learning movies were presented, in which the cow always took the same of the two paths, so participants could learn about the cow’s “usual” behaviour. Whether the cow was taking the lower or the upper path in the learning trials was counterbalanced between participants, resulting in two conditions. In condition 1, the cow took the lower path in the learning trials and in condition 2 the cow always took the upper path. Then, the interruption of the respective path was presented, followed by the three test movies.

### Measures

The Tobii Studio IV-T fixation filter was used. It consisted of a maximum gap length of 75 ms, eyes’ angular velocity within a 20-ms time interval and a velocity threshold of 30 degrees/second. Adjacent fixations were merged to a maximum of 75 ms between fixations and a maximum angle of 0.5 degrees. Minimum fixation duration was 60 ms. In order to analyse participants’ eye gazes, and in line with previous research (e.g. Daum et al. 2012; Falck-Ytter et al. 2006), two areas of interest (AOI, each covering 4.54% of the screen; see also Fig. 1d) were situated on the sections where the paths reappeared from the occluder. A third AOI covered the whole screen (100%) to control for missing data in the other two AOIs. In test trials, participants’ gaze behaviour was measured from the moment the cow completely disappeared behind the occluder until the end of the movie (4.63 s). To be included into analysis, participants had to show eye-gaze data (i.e. fixations towards the screen) in at least two of the three test trials. In learning trials, gaze behaviour was measured from the time the cow disappeared behind the occluder until it reappeared (1.29 s for condition 1 and 1.49 s for condition 2). Three different measures, a frequency score, a first fixation score and a differential looking score (DLS) were used to analyse participant’s gaze behaviour in test trials (see below). For analysis of the learning trials, only the differential looking score was used, as it is better suited when looking at individual trials.

*Frequency of Anticipations.* A score was generated to see whether all age groups show an equal amount of anticipations in all three test trials to either the continuous or interrupted path. Thus, anticipation to either of the paths was coded with 1. If participants did not show any anticipation to either of the paths but fixated somewhere else on the screen, this was coded with 0 (Ganglmayer et al. 2019a, b).

*First Fixation Score.* To assess whether participants fixated first the upper or lower path after the cow’s disappearance, a first fixation score was generated (see, e.g. Paulus et al. 2011). For test trials, participants’ gaze behaviour was coded with 1 when they fixated the continuous path first and with 0, when they fixated the interrupted path first. If they did not fixate on either of the two AOIs or did not show any fixation to the screen during the anticipatory period, this was treated as a missing value.

*Differential Looking Score (DLS).* To investigate whether participants spent more time on one AOI in relation to the other, a DLS was calculated (see, e.g. Senju et al. 2009). This score allows controlling for corrective eye movements, as participants could look first to one AOI but fixate the other AOI longer in total. Thus, the total looking time to the AOI of the interrupted path was subtracted from the total looking time to the AOI of the continuous path, divided by the sum of overall total looking time to both AOIs. Similarly, for learning trials, the total looking time to the AOI of the “other path” was subtracted from the total looking time to the AOI of the path the cow always took in the learning trials, divided by the sum of total looking time to both AOIs.

IBM SPSS Statistics 24 (SPSS Inc., Chicago, IL, USA) was used for the statistical analyses. To assess the learning behaviour of participants in the learning trials, a regression coefficient analysis was performed with the DLS (Lorch and Myers 1990). Thus, a regression for each participant was calculated over the five learning trials and the slope of the regression was extracted for further analysis. (The intercept was not used for further analysis, since participants could not have an expectation of the agent’s action in the first trial.) A one-way ANOVA with the between-subject factor age group was performed to see whether the age groups differ from each other in their learning performance. Further, a one-sample t-test was performed, to see whether the slope was significantly different from zero and would thus indicate learning performance over the trials. Further, for the DLS, in total 3.09% of the gaze data was missing in the learning trials. For these cases, the mean of the respective age group for that trial was inserted.

To analyse whether participants show an equal amount of general anticipations over the three test trials, a general estimating equations model (GEE; Zeger and Liang 1986) was performed with the predictor variables age group, trial and the interaction of age group and trial. Another general estimating equations model was performed for analysis of the First Fixation Score of the test trials, with the same predictors, namely age group, trial and interaction of age group and trial. Furthermore, Chi-square tests were calculated for each age group and test trial, to see whether participants’ performance differs from chance level.

For analysis of the DLS of test trials a mean over the three test trials was generated (e.g. Schuwerk and Paulus 2016) and a one-way ANOVA with the between-subject factor age group (2-year-olds, 5-year-olds, younger and older adults) was performed. Further one-sample t-tests were calculated for each age group separately, to see whether participants’ looking bias differed from chance level. For test trials, missing values were not replaced, since these scores were averaged over the three test trials. To further analyse how participants perform over the three test trials, a mixed ANOVA with trial and age group as a factor was conducted for the DLS.

## Results

### Learning trials

According to the one-way ANOVA, there is no significant difference of the slopes between the four age groups, *F*(3, 177) = 0.517, *p* = 0.671, *η*_*p*_^2^ = 0.01, indicating that all four age groups showed no significant differences in their performance in the learning trials. To see whether their overall performance is significantly different from chance level, a one-sample t-test against zero was performed for the slope and turned out significant with *t*(180) = 6.33,* p* < 0.001, *M* = 0.10, *SD* = 0.22. This indicates that over all age groups is a trend for learning the agent’s path choice in the learning trials.

### Test trials

*Frequency of Anticipations.* Participants anticipated in 448 out of 543 trials (82.5%). A generalized estimating equations model with an unstructured working correlation matrix, logit link function and binomial distribution (GEE; Zeger and Liang 1986) demonstrated that neither of the predictors (age group and trial, as well as an interaction of age group and trial) had an influence on participants’ anticipations. All four age groups anticipated equally over all three test trials. Results of the generalized estimating equations model can be found in Table 1.

**Table 1:** Results of the generalized estimating equations model with the predictors age group, trial and an interaction of age group and trial on the frequency of anticipations

Predictor	B	SE	Wald	*df*	*p* value	Exp(B)	95% confidence interval for Exp(B)	
							Lower	Upper
Age group	0.003	0.32	0.00	1	0.993	1.00	0.53	1.89
Trial	−0.37	0.34	1.19	1	0.276	0.69	0.36	1.34
Age group*Trial	−0.024	0.12	0.04	1	0.840	0.97	0.77	1.23

*First Fixation Score.* The general estimating equations model with an unstructured working correlation matrix, logit link function and binomial distribution (GEE; Zeger and Liang 1986) showed that only the predictor of age group had a significant effect on the First Fixation Score. In sum, 2-year-olds made 22 anticipations to the continuous path and 74 to the interrupted path. The 5-year-olds made 42 anticipations to the continuous and 86 to the interrupted path. In contrast, younger adults anticipated towards the continuous path 73 times and 34 times to the interrupted path. Similarly, older adults showed 86 anticipations to the continuous and 31 anticipations to the interrupted path. Detailed results of the GEE can be seen in Table 2.

**Table 2:** Results of the generalized estimating equations model with the predictors age group, trial and an interaction of age group and trial on the First Fixation Score

Predictor	B	SE	Wald	*df*	*p* value	Exp(B)	95% confidence interval for Exp(B)	
							Lower	Upper
Age group	1.04	0.27	14.86	1	<0 .001	2.84	1.67	4.82
Trial	0.25	0.30	0.68	1	0.410	1.28	0.71	2.32
Age group*Trial	−0.11	0.12	0.83	1	0.363	0.90	0.72	1.13

To see whether participants’ performance differs from chance level, Chi-square tests were conducted for each age group and test trial separately. Results can be seen in Table 3. The tests were all significant, except for the 2-year-olds, 5-year-olds and younger adults for the third test trial, which indicates that for these age groups their performance is at chance level in the third test trial.

**Table 3:** Results of the Chi-square tests of the First Fixation Score for each test trial and age group

	Test trial 1	Test trial 2	Test trial 3
2-year-olds	*χ*^*2*^(1) = 11.77, *p* = .001	*χ*^*2*^(1) = 14.24, *p* < .001	*χ*^*2*^(1) = 3.57, *p* = .059
5-year-olds	*χ*^*2*^(1) = 6.15, *p* = .013	*χ*^*2*^(1) = 6.10, *p* = .014	*χ*^*2*^(1) = 3.10, *p* = .078
Younger adults	*χ*^*2*^(1) = 11.31, *p* = .001	*χ*^*2*^(1) = 5.44, *p* = .020	*χ*^*2*^(1) = 0.50, *p* = .480
Older adults	*χ*^*2*^(1) = 8.81, *p* = .003	*χ*^*2*^(1) = 7.41, *p* = .006	*χ*^*2*^(1) = 9.76, *p* = .002

We further calculated for each test trial a Chi-square test to see whether the differences between the age groups are significant with this score as well. Results revealed a significant result for the first test trial with *χ*^*2*^(3) = 37.98, *p* < 0.001, for the second test trial with *χ*^*2*^(3) = 32.93, *p* < 0.001 and for the third test trial with *χ*^*2*^(3) = 16.91, *p* = 0.001, indicating a significant effect of age group for all three test trials.

**Fig.2:**
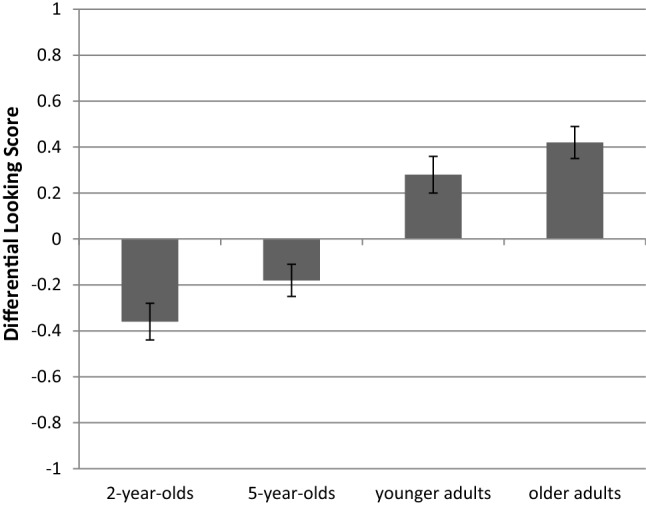
Descriptives for the Differential Looking Score (DLS) of the test trials for each age group. Error bars represent the standard errors of the means

*Differential Looking Score (DLS).* The one-way ANOVA with the mean over the three test trials yielded a significant effect of age group, with *F*(3, 177) = 23.98, *p* < 0.001, η_p_^2^ = 0.29. Bonferroni-adjusted pairwise comparisons resulted in the same pattern as for the first fixation score. Two-year-olds (*M* = – 0.36, *SE* = 0.08) and 5-year-olds (*M* = – 0.18, *SE* = 0.07) did not significantly differ from each other, with *p* = 0.559, Cohen’s *d* = 0.34. There was also no significant difference between younger (*M* = 0.28, *SE* = 0.08) and older adults (*M* = 0.42, *SE* = 0.07), *p* = 1.000, Cohen’s *d* = 0.29. However, both the 2- and 5-year-olds differed from the younger and older adults, all *p* < 0.001. Further, the means of all age groups were significantly different from chance level, with the 2-year-olds [*t*(41) = – 4.34, *p* < 0.001] and 5-year-olds [*t*(46) = – 2.42, *p* = 0.020] showing a looking bias towards the interrupted path (see also Fig. 2), and the younger adults [*t*(44) = 3.71, *p* = 0.001] and older adults [*t*(46) = 6.12, *p* < 0.001] towards the continuous path.

**Fig.3:**
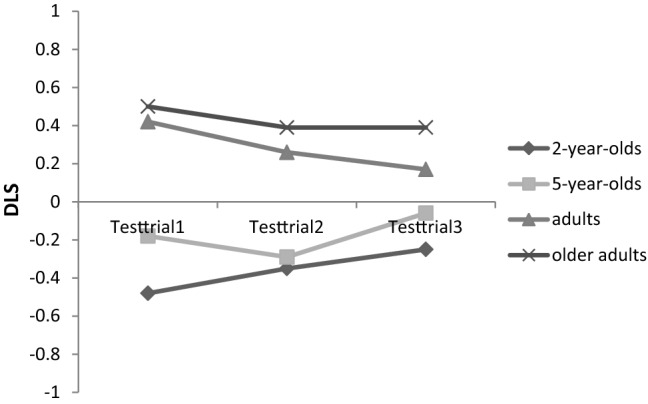
Descriptives of the Differential Looking Score (DLS) for each test trial and age group

The trial-by-trial analysis with the mixed ANOVA yielded a significant main effect of age, with *F*(3, 166) = 22.81, *p* < 0.001, η_p_^2^ = 0.29, as well as a significant interaction effect of age and trial, with *F*(6, 332) = 2.18, *p* = 0.046, η_p_^2^ = 0.04. There was no main effect of trial, *F*(2, 332) = 1.15, *p* = 0.318, η_p_^2^ = 0.01. To investigate the significant interaction effect further, post hoc one-way ANOVAs with the within-subject factor trial were calculated for each age group separately. Only the ANOVA for the younger adults yielded a significant effect of trial with *F*(2, 86) = 3.16, *p* = 0.047, η_p_^2^ = 0.07. Pairwise comparisons demonstrated that the looking bias in trial 3 (*M* = 0.15, *SE* = 0.10) differs significantly from trial 1 (*M* = 0.42, *SE* = 0.09; *p* = 0.044), indicating a significant decrease in the looking bias from trial 1 to trial 3 (see also Fig. 3 for all descriptives). All other pairwise comparisons were not significant. The ANOVA for the 2-year-olds was not significant with *F*(2, 68) = 1.45, *p* = 0.241, η_p_^2^ = 0.04, as well as for the 5-year-olds with *F*(2, 86) = 2.36, *p* = 0.101, η_p_^2^ = 0.05, and older adults with *F*(2, 92) = 0.77, *p* = 0.465, η_p_^2^ = 0.02. For the descriptives, see as well Fig. 3.

### Control measure

*MMSE.* We observed no indications of beginning dementia within the age group of older adults (*M* = 29.11, *SD* = 1.10, range = 26–30). Further, there were no significant correlations between the MMSE and the First Fixation Score (*r* = – 0.01, *p* = 0.937) or the DLS (*r* = – 0.05, *p* = 0.733).

### Additional analysis

To exclude the possibility that children’s looking bias towards the interrupted path in test trials is a result of their failure to recognize and process the gap in the blocking movie, an additional correlational analysis was performed. Correlations between the First Fixation Score resp. DLS of the test trials and participants’ looking time towards the interrupted path during the blocking movie were calculated. An AOI was defined around the area of the gap (11.05%), and the total looking time towards this area was measured from the start of the gap’s appearance until the end of the blocking movie (i.e. 16.5 s in total). Results yielded no significant correlation between the total looking time towards the gap, and the First Fixation Score and DLS for any of the four age groups (all *p*’s > 0.151). This indicated that participants’ anticipatory looking behaviour in test trials was not related to the time they spent looking at the gap during the blocking movie.

Another additional analysis was performed to rule out the possibility that children’s anticipations to the interrupted path might be driven by the salience of the gap and might thus be a result of bottom-up processes rather than a result of their previous observation. If this would be the case, children should look longer towards the gap during the anticipatory period or look at the gap first before looking towards the interrupted path. Details of this analysis (method and results) can be found in supplemental material. Results suggest that participants, who anticipated towards the interrupted path, did not necessarily look at the gap (first) during the anticipatory time period. In fact, results showed that they first made their anticipation towards the interrupted path. If anything they—on average—looked at the gap after their anticipation to the interrupted path.

## Discussion

The current study investigated whether humans integrate situational constraints flexibly when anticipating others’ actions. To this end, 2-year-olds, 5-year-olds, younger and older adults observed an agent repeatedly taking one of two paths to reach a goal. Then, this path became blocked. We assessed by means of eye-tracking whether participants subsequently anticipated the agent to take the other, passable path. This would indicate that participants integrated the situational constraints in their anticipation behaviour. Results revealed that younger and older adults integrated the situational constraints flexibly in their action anticipations. This demonstrates the informative power of context information, as suggested by predictive coding theories (Clark 2013a). Moreover, our analyses revealed clear age-related differences: 2- and 5-year-olds anticipated towards the interrupted path and did not integrate the situational constraints in their anticipations. We discuss these findings further in the next sections.

According to our results, 2- and 5-year-olds relied on the agent’s previous observed behaviour and thus anticipated towards the interrupted path. They did not integrate the situational constraints flexibly when anticipating the agent’s action. Thus, our results are not in line with claims that young children consider situational constraints from early on (Gergely and Csibra 2003). They rather suggest that a prior, based on the agent’s previous observed behaviour, dominantly influences children’s anticipations (Daum et al. 2012; Ganglmayer et al. 2019b). These results also fit well with the observation of Paulus et al. (2011) who showed that infants (in contrast to adults) did not adapt their anticipations towards contextual changes, even after they have repeatedly observed the changed behaviour of the agent. So even if infants have seen that the agent performed an alternative behaviour due to the context change, they did not change their anticipations. This suggests that, in line with our results, statistical learning is a prevailing mechanism within infants and children when anticipating others’ actions (Ruffman 2014).

Interestingly, we further observed no significant differences in performance between the 2- and 5-year-olds. This was surprising, since we expected that 5-year-olds would perform better than 2-year-olds following results of previous studies that suggested advanced anticipation abilities of more complex actions from 3 years onwards (Daum et al. 2012; Paulus et al. 2017). Furthermore, prior studies have demonstrated that already infants process contextual changes and adapt their anticipations to changing context information (e.g. Adam et al. 2016, 2017; Ambrosini et al. 2015; Kanakogi and Itakura 2011). It could be argued that specificities of the chosen paradigm made it difficult for children to include the situational constraints in their action anticipations. For example in this set-up, a paradigm with an occluder was chosen, in order to elicit anticipatory eye movements. One could argue that the occlusion of the action could have also posed high cognitive demands on children, such as attention or working memory and therefore complicated the inclusion of situational constraints for children. Yet, current empirical evidence suggests that the use of an occluder might not even significantly influence 12-month-old infants when anticipating other’s actions (see Ganglmayer et al. 2019b). Moreover, 5-year-old children possess quite sophisticated representational abilities as evidenced in, for example, Theory of Mind (Wimmer and Perner 1983). Thus, it seems unlikely that a short occlusion of an action might be that cognitively challenging for them.

Importantly, it should be considered that the focus of the current study are visual anticipations. These are (in contrast to verbal predictions) fast and might rely on simple, rather automatic processes (e.g. Apperly and Butterfill 2009). It seems likely that the consideration of situational constraints is cognitively effortful and might rely on rather slow processes that are still developing in young children. Indeed, a recent study exploring explicit (that is, verbal) action prediction showed that by 4–5 years, but not at 3 years children consider efficiency when predicting others’ actions (Gönül and Paulus, in press). Importantly, a dissociation between more sophisticated verbal action prediction and less sophisticated anticipatory gaze was also reported by Schuwerk and Paulus (2016). We will discuss this possibility also below in relation to the results of the pilot study.

In contrast, younger and older adults flexibly integrated the occurred situational constraints when anticipating others’ actions. They show flexibility in their anticipations as they have previously learned about the agent’s action through repeated observation, but changed their anticipations flexibly due to contextual changes. This provides evidence for context sensitivity during action anticipation. This is in line with predictive coding theories (Clark 2013a, 2013b; Kilner et al. 2007). Our results support the assumption that context-informed priors have a significant influence on action processing. Even if adults already have expectations about an agent’s behaviour based on other priors (e.g. previous observation), they use context information to update these expectations. They have previously learned that an agent performs an action in a certain way (the cow always taking one specific path) and can flexibly change their predictions based on new and more reliable information (i.e. the context change). This suggests that adults integrate multiple information sources when anticipating others’ actions and also weigh the information sources in accordance with their reliability (see also Ambrosini et al. 2015).

Within predictive coding theory, it has been claimed that this ability, to weigh different information sources according to their predictive power, is based on hyperpriors. In the current study, participants are confronted with two different information sources/priors to predict the agent’s behaviour. One is the agent’s previous behaviour (it always takes one specific path), and the other one is the situational constraint (this path becomes blocked and only the other one is passable). Now the two priors have to be integrated and weighted according to their precision: Which one is the most reliable and should thus be used for predictions? It has been claimed that this kind of higher-level knowledge (i.e. hyper priors) is not fixed, but must be built and learned through experience (Clark 2013a).

Furthermore, our results did not reveal a decrease in anticipation abilities for older adults. Thus, our results do not support the claim that action anticipation declines at older age (Diersch et al. 2012, 2016). Although even healthy older adults are likely to show decline in several executive functions (e.g. Gazzaley et al. 2008), this does obviously not affect their ability to integrate situational constraints in action anticipations for a simple situation as in our task. However, these interpretations are speculative since executive functions were not directly assessed. Future studies are needed to see whether there is a relation between executive control processes and the ability to integrate changing context information into action anticipations.

Interestingly, results revealed that the looking behaviour of younger adults changed over the three test trials. More precisely, their looking bias towards the continuous path declines in the last trial towards chance level. Importantly, adults showed a looking bias towards the continuous path in the first test trials, indicating that a mature cognitive system can update its priors in relation to occurred situational constraint. The decline of their looking bias towards chance level in the last test trial might thus be a result of the agent never reappearing from the occluder during test trials. Thus, participants do not gain any feedback on how the cow’s behaviour might change due to the occurred situational constraints. On the one hand, this might indicate that the informativeness of the prior of the situational constraint declines over time. Predictive coding theory does not give any clear indication about the scope of prior information (see also Tewolde et al. 2017). Our results could suggest that a prior based on situational constraints might only work on a very short time scale, especially when there is no additional feedback on whether an agent takes the specific information into account or not. On the other hand, a decline of the looking bias towards the continuous path in the last trial within younger adults might also be due to motivational reasons. Since older adults kept anticipating towards the continuous path also in the last test trial, it could be that this age group was simply more motivated than the younger adults. Future studies are needed to explore these possibilities in greater detail.

Importantly, in an additional pilot task without any learning phase (see methods section), we also explicitly asked 3 to 6-year-old children, which path the agent could take after the gap appeared on one of the paths. This pilot task was designed to test whether children “understood” the paradigm and that the agent cannot walk across the gap. Notably, this task differs from the actual study in some aspects, as it did not include any of the learning trials. Children between 3 and 6 years of age observed the cow once taking the upper and once taking the lower path before one of the paths became interrupted. Most of the children explicitly referred to the continuous path. Thus, it seems very unlikely that children did not understand the paradigm or that the agent cannot walk across the interrupted path. Given reduced language abilities, this additional task could not be administered to 2-year-old children. While we cannot assume with certainty that also 2-year-olds understood the stimuli, we can assume in the light of the pilot results that 5-year-olds clearly understood them. Given that in our main task 5-year-olds nevertheless showed the same pattern of results as the 2-year-olds, it renders unlikely that the results of the 2-year-olds could be solely explained by problems in understanding the stimuli. Nevertheless, future studies could investigate whether the same underlying mechanisms were responsible for the same gaze patterns of the 2- and 5-year-olds.

Interestingly, the results of the pilot task indicate that 3- to 6-year-old children include situational constraints when they are explicitly asked to give a verbal answer about their expectation of the agent’s upcoming action. This pattern of results (better performance in verbal reasoning tasks compared to visual anticipations) parallels previous findings (e.g. Gönül and Paulus, in press; Schuwerk and Paulus 2016). For example, Gönül and Paulus (in press) showed across five studies that 4- to 5-year-olds, but not 3-year-olds consider efficiency when verbally predicting or reasoning about others’ actions. This difference between explicit and implicit information processing relates to proposals on the existence of two systems for the processing of social information (Apperly and Butterfill 2009; Strack and Deutsch 2004). Our findings might suggest that children first learn to consider situational constraints on an explicit level and only later in development, with increasing automatization use this knowledge on an implicit level to visually predict other’s actions (Schuwerk and Paulus 2016; Paulus et al. 2017). Nevertheless, we do not know whether they flexibly include situational constraints in their verbal predictions if they had previously been presented with a learning phase: If they have previously seen for several times that the agent always takes one instead of the other path. When interpreting the results, one has to be aware of the differences between the actual study and the pilot task. Further studies with comparisons of more similar designs might be interesting to see whether children include situational constraints in their verbal predictions, when they have previously been presented with a statistical prior, i.e. the agent taking repeatedly one of the paths instead of the other.

Furthermore, our results revealed that there were no significant relations between the looking time towards the gap and the children’s anticipations. Thus, our results do not imply that children simply did not look at the gap long enough to process its appearance and therefore kept anticipating towards the interrupted path. Accordingly, it seems very unlikely that children did not recognize the gap or did not understand that the gap is not passable.

One could also argue that participants may think that the cow doesn’t know about the appearance of the impassable gap. However, on the one hand, the cow gives clear, verbal indications that she has seen the gap and states “ah now it’s not passable anymore”, and on the other hand there is empirical evidence that by 24 months of age, children already acquired level 1 perspective taking (Moll and Tomasello 2006; 2007). Further, the results of the pilot study underline this argumentation.

Moreover, results of a further additional analysis suggest that children’s anticipations towards the interrupted path were not bottom-up driven by the salience of the gap. In particular, results showed that in test trials children first looked towards the interrupted path and if anything they—on average—looked at the gap after their anticipation to the interrupted path. This indicates that children made an anticipation based on their previous observation. However, results of this analysis have to be interpreted carefully, as the visual angle might encompass a larger area. That is, since the angle of vision covers a larger area than just the fixation point, we cannot exclude the possibility that the gap was in the area of vision when fixating the interrupted path, and thus, this alternative cannot be completely eliminated. Future research has to address this point in order to explore this alternative hypothesis in greater detail.

Last but not least, analyses of the learning trials suggest a comparable learning performance for all four age groups over the learning phase, since there was no difference between the age groups in their learning performance. This demonstrates that children learned about the agent’s path preference and diminishes the possibility that the differences between the age groups in test trials are based on differences in previous learning performance.

## Limitations and open questions

Although the age groups included in the current study were selected on the basis of thorough theoretical considerations, future studies could examine further age groups between five years of age and (early) adulthood. This would be helpful to learn more about the underlying mechanisms of the ability to flexibly integrate contextual information into action anticipations.

Also, as described earlier, predictive coding theories distinguish between the integration of several information sources and the weighing of these information sources according to their reliability. In point of this view, it is still not clear whether children have problems with the integration of the information or with the weighing (or both). It could be possible that they have problems with the synchronous integration of the contextual changes and the agent’s previous behaviour, or that they have problems “deciding” that the change in context is more predictive than the agent’s previous behaviour. Predictive coding theories do not offer any suggestions concerning the developmental trajectory of a “successful” predictive system. Further theoretical and empirical insights are needed.

In line with this, it would be interesting to assess directly the relationship between the individual capabilities in critical cognitive functions, such as working memory, inhibitory control (inhibiting the prior of the agent’s previous behaviour in order to make a prediction in relation to the contextual changes) or cognitive flexibility and the ability to flexibly integrate contextual changes in action anticipations. This could improve our knowledge on the contribution of these executive functions in action anticipation.

In the beginning of the procedure, participants were familiarized with the whole set-up. They were provided with the information that the agent usually walks on the paths and not on the green surface. Likewise, during the learning trials participants were presented with the protagonist exclusively walking on the paths and not on the green surface. Additionally, a pilot study was conducted to make sure that participants understood the whole set-up and that the agent cannot cross the gap. However, future studies could use different types of familiarization trials and checks, to make sure that the whole set-up is clear and unambiguous for participants.

In the same vein, it might be interesting to manipulate the strength of a prior in future studies, in order to see how a stronger prior might influence participant’s looking behaviour. For example, one could include an additional learning episode that shows the cow actively avoiding the green gap.

One could argue that saliency effects might have affected our results. The interrupted path had to be—by definition—visually different from the continuous path. This difference had to be clear also for our younger participants. Thus, as a possible alternative explanation it could be argued that children’s anticipations towards the interrupted path were actually a result of bottom-up processes due to the salience of the gap and not because of their previous observation. However, several aspects speak against this interpretation. First, even if children’s anticipations were driven by the saliency of the interrupted path, this would indicate that their conceptual knowledge was not consolidated enough to overcome this low-level visual cue and they thus anticipated towards the interrupted path (in contrast to adults). This still indicates that there are age-related differences in the consideration of situational constraints in visual anticipations. Second, we administered more than one test trial. Saliency effects are subjected to habituation and their impact should thus be less powerful in repeated presentation of the same trial (e.g. Donk and Soesman 2010). Yet, we did not observe clear changes across trials for children. Third, we did not only analyse participants’ first look towards one of the two paths, but also calculated the DLS, which includes a longer time frame (see also Donk and Soesman 2010). That is, even if children’s attention was first captured by a potentially more salient interrupted path, this could have been balanced out by the DLS. Last but not least, an additional analysis was performed (see supplemental material), which showed that participants who anticipated towards the interrupted path did not necessarily also look towards the gap. On average they looked at the path first and if anything they fixated the gap afterwards. This indicates that children’s anticipations towards the interrupted path were not driven by the salience of the gap. Given these aspects, this alternative explanation seems very unlikely.

Even though the current research question was answered by using an established paradigm with an animated non-human agent (e.g. Daum et al. 2012; Biro 2013; Ganglmayer et al. 2019a, b; Hamlin et al. 2007; Liu et al. 2017), it would be interesting to explore whether similar or different results would be obtained when using a human agent. While Ganglmayer et al. (2019a, b) found no differences in the overall pattern of goal anticipation for human and non-human animated agents, others observed that cues of agency can elicit infants’ anticipations (e.g. Adam and Elsner 2018; Falck-Ytter et al. 2006). Although the agent in this study shows clear cues of agency, such as having a face, a voice, self-propelledness and interacts with another agent, future studies could investigate whether young children include situational constraints differently or earlier when anticipating actions of human agents.

## Conclusion

In sum, our results suggest that adults do not only take situational constraints into account when anticipating another’s action, but that they also change their previously acquired expectations of another’s behaviour due to the contextual change. This indicates that context information is taken into account and is thus in line with claims from predictive coding theories (Clark 2013a). However, 2- and 5-year-olds did not integrate the contextual changes in their anticipations, suggesting that this ability develops later in childhood.

## Supplementary Information

Supplementary information

## References

[CR1] Adam M, Reitenbach I, Papenmeier F, Gredebäck G, Elsner C, Elsner B (2016). Goal saliency boosts infants’ action prediction for human manual actions, but not for mechanical claws. Infant Behav Dev.

[CR2] Adam M, Reitenbach I, Elsner B (2017). Agency cues and 11-month-olds’ and adults’ anticipation of action goals. Cognit Dev.

[CR3] Adam M, Elsner B (2018). Action effects foster 11-month-olds’ prediction of action goals for a non-human agent. Infant Behav Dev.

[CR68] Ambrosini E, Costantini M, Sinigaglia C (2011). Grasping with the eyes. J Neurophysiol.

[CR4] Ambrosini E, Reddy V, De Looper A, Costantini M, Lopez B, Sinigaglia C (2013). Looking ahead: anticipatory gaze and motor ability in infancy. PLoS ONE.

[CR5] Ambrosini E, Pezzulo G, Costantini M (2015). The eye in hand: Predicting others' behavior by integrating multiple sources of information. J Neurophysiol.

[CR6] Apperly IA, Butterfill SA (2009). Do humans have two systems to track beliefs and belief-like states?. Psychol Rev.

[CR7] Bekkering H, De Bruijn ER, Cuijpers RH, Newman-Norlund R, Van Schie HT, Meulenbroek R (2009). Joint action: Neurocognitive mechanisms supporting human interaction. Top Cognit Sci.

[CR8] Biro S (2013). The role of the efficiency of novel actions in infants’ goal anticipation. J Exp Child Psychol.

[CR11] Clark A (2013). Whatever next? Predictive brains, situated agents, and the future of cognitive science. Behav Brain Sci.

[CR12] Clark A (2013). The many faces of precision (Replies to commentaries on “Whatever next? Neural prediction, situated agents, and the future of cognitive science”). Front Psychol.

[CR13] Csibra G, Gergely G, Biró S, Koós O, Brockbank M (1999). Goal attribution without agency cues: the perception of “pure reason” in infancy. Cognition.

[CR14] Daum MM, Attig M, Gunawan R, Prinz W, Gredebäck G (2012). Actions seen through babies’ eyes: a dissociation between looking time and predictive gaze. Front Psychol.

[CR15] Diersch N, Cross ES, Stadler W, Schütz-Bosbach S, Rieger M (2012). Representing others’ actions: the role of expertise in the aging mind. Psychol Res.

[CR16] Diersch N, Jones AL, Cross ES (2016). The timing and precision of action prediction in the aging brain. Hum Brain Mapp.

[CR17] Donk M, Soesman L (2010). Salience is only briefly represented: evidence from probe-detection performance. J Exp Psychol Hum Percept Perform.

[CR18] Eshuis R, Coventry KR, Vulchanova M (2009). Predictive eye movements are driven by goals, not by the mirror neuron system. Psychol Sci.

[CR19] Falck-Ytter T, Gredebäck G, von Hofsten C (2006). Infants predict other people's action goals. Nat Neurosci.

[CR20] Faul F, Erdfelder E, Buchner A, Lang A-G (2009). Statistical power analyses using G*Power 3.1: tests for correlation and regression analyses. Behav Res Methods.

[CR21] Flanagan JR, Johansson RS (2003). Action plans used in action observation. Nature.

[CR22] Folstein MF, Folstein SE, McHugh PR (1975). ‘‘Minimental state’’: a practical method for grading the cognitive state of patients for the clinician. J Psychiatr Res.

[CR23] Ganglmayer K, Schuwerk T, Sodian B, Paulus M (2019a) Do children and adults with autism spectrum condition anticipate others’ actions as goal-directed? A predictive coding perspective*.* J Autism Dev Disorders. 10.1007/s10803-019-03964-8 10.1007/s10803-019-03964-8 30850911

[CR24] Ganglmayer K, Attig M, Daum MM, Paulus M (2019). Infants’ perception of goal-directed actions: a multi-lab replication reveals that infants anticipate paths and not goals. Infant Behav Dev.

[CR25] Gampe A, Daum MM (2014). Productive verbs facilitate action prediction in toddlers. Infancy.

[CR26] Gazzaley A, Clapp W, Kelley J, McEvoy K, Knight RT, D’Esposito M (2008). Age-related top-down suppression deficit in the early stages of cortical visual memory processing. PNAS.

[CR27] Gergely G, Csibra G (2003). Teleological reasoning in infancy: the naıve theory of rational action. Trends Cognit Sci.

[CR28] Gergely G, Nádasdy Z, Csibra G, Bíró S (1995). Taking the intentional stance at 12 months of age. Cognition.

[CR29] Gönül G, Paulus M (in press) Children’s reasoning about the efficiency of others’ actions: The development of rational action prediction. Journal of Experimental Child Psychology. 10.1016/j.jecp.2020.105035 33341019

[CR31] Gredebäck G, Stasiewicz D, Falck-Ytter T, Rosander K, von Hofsten C (2009). Action type and goal type modulate goal-directed gaze shifts in 14-month-old infants. Dev Psychol.

[CR33] Henrichs I, Elsner C, Elsner B, Wilkinson N, Gredebäck G (2014). Goal certainty modulates infants’ goal-directed gaze shifts. Dev Psychol.

[CR34] Hohwy J, Roepstorff A, Friston K (2008). Predictive coding explains binocular rivalry: an epistemological review. Cognition.

[CR35] IBM SPSS Statistics (24) [computer software]. Chicago, IL: SPSS Inc.

[CR36] Kanakogi Y, Itakura S (2011). Developmental correspondence between action prediction and motor ability in early infancy. Nat Commun.

[CR38] Kilner JM, Friston KJ, Frith CD (2007). Predictive coding: an account of the mirror neuron system. Cogn Process.

[CR39] Kochhann R, Varela JS, de Macedo Lisboa CS, Chaves MLF (2010). The mini mental state examination: review of cutoff points adjusted for schooling in a large Southern Brazilian sample. Dementia Neuropsychol.

[CR41] Liu S, Ullman TD, Tenenbaum JB, Spelke ES (2017). Ten-month-old infants infer the value of goals from the costs of actions. Science.

[CR43] Moll H, Tomasello M (2006). Level 1 perspective-taking at 24 months of age. Br J Dev Psychol.

[CR42] Moll H, Tomasello M (2007). How 14-and 18-month-olds know what others have experienced. Dev Psychol.

[CR44] Oliva A (2005) Gist of the scene. In: Neurobiology of attention, Academic Press, Cambridge, pp 251–256. https://doi.org/10.1016/B978-012375731-9/50045-8

[CR45] Paulus M (2012). Action mirroring and action understanding: An ideomotor and attentional account. Psychol Res.

[CR46] Paulus M, Hunnius S, van Wijngaard C, Vrins S, van Rooij I, Bekkering H (2011). The role of frequency information and teleological reasoning in infants' and adults' action prediction. Dev Psychol.

[CR47] Paulus M, Schuwerk T, Sodian B, Ganglmayer K (2017). Children’s and adults’ use of verbal information to visually anticipate others’ actions: a study on explicit and implicit social-cognitive processing. Cognition.

[CR48] Ruffman T (2014). To belief or not belief: children’s theory of mind. Dev Rev.

[CR49] Ruffman T, Taumoepeau M, Perkins C (2012). Statistical learning as a basis for social understanding in children. Br J Dev Psychol.

[CR51] Schuwerk T, Paulus M (2016). Preschoolers, adolescents, and adults visually anticipate an agent's efficient action; but only after having observed it frequently. Quart J Exp Psychol.

[CR52] Sebanz N, Knoblich G (2009). Prediction in joint action: What, when, and where. Top Cognit Sci.

[CR53] Senju A, Southgate V, White S, Frith U (2009). Mindblind eyes: an absence of spontaneous theory of mind in Asperger syndrome. Science.

[CR54] Skerry AE, Carey SE, Spelke ES (2013). First-person action experience reveals sensitivity to action efficiency in prereaching infants. Proc Natl Acad Sci.

[CR56] Stapel JC, Hunnius S, Bekkering H (2012). Online prediction of others’ actions: the contribution of the target object, action context and movement kinematics. Psychol Res.

[CR57] Strack F, Deutsch R (2004). Reflective and impulsive determinants of social behavior. Personal Soc Psychol Rev.

[CR58] Tewolde FG, Bishop DVM, Manning C (2017). Visual motion prediction and verbal false memory performance in autistic children. Autism Res.

[CR59] Van Overwalle F (2010). Infants' teleological and belief inference: A recurrent connectionist approach to their minimal representational and computational requirements. NeuroImage.

[CR61] von Hofsten C, Kochukhova O, Rosander K (2007). Predictive tracking over occlusions by 4-month-old infants. Dev Sci.

[CR62] Wermelinger S, Gampe A, Daum MM (2019). The dynamics of the interrelation of perception and action across the life span. Psychol Res.

[CR64] Wurm MF, Schubotz RI (2012). Squeezing lemons in the bathroom: contextual information modulates action recognition. Neuroimage.

[CR66] Zeger SL, Liang KY (1986). Longitudinal data analysis for discrete and continuous outcomes. Biometrics.

[CR67] Zelazo PD, Craik FI, Booth L (2004). Executive function across the life span. Acta Physiol (Oxf).

