# Supplementary material for: Adults, but not preschoolers or toddlers integrate situational constraints in their action anticipations: a developmental study on the flexibility of anticipatory gaze
Source: Cogn Process. 2021 Mar 24;22(3):515–28. doi: 10.1007/s10339-021-01015-8 (PMC8324589; doi:10.1007/s10339-021-01015-8)
Supplement: Supplementary file 1 — Supplementary information [file 10339_2021_1015_MOESM1_ESM.pdf]

Supplemental material

Additional analysis:

An alternative interpretation concerns that 2- and 5-year-old's gaze behavior towards the interrupted path could be driven by the saliency of the gap and thus might be a result of bottom-up processes, additional post-hoc analyses of participant's gaze behavior were performed to rule out this possible alternative explanation. Thus, gaze behavior in all three test trials of those participants was analyzed, who showed a first anticipation towards the interrupted path in the original analysis (*First Fixation Score*; participants who showed a first anticipation towards the continuous path or did not show any anticipation at all were excluded from this analysis). If children's anticipatory looking behavior towards the interrupted path was mainly driven by the gap rather than the path, one would expect that children would focus longer on the gap than the path or look at the gap first before they look towards the path. Hence, two different measures were used to analyze participant's gaze behavior after the agent disappeared behind the occluder in the test trials: First, it was analyzed whether participants actually fixated the AOI of the gap *before* they fixated the AOI of the interrupted path (*First Fixation*). Chi-Square-Tests were conducted between the number of first fixations towards the path and the number of first fixations towards the gap. Tests were calculated for each age group over all three test trials and for each test trial over all three age groups. Second, the total looking time towards the AOI of the gap and the total looking time towards the AOI of the interrupted path (*Total Looking Time*) were compared within participants, to see whether participants –in sum- looked longer to the gap than to the path. Thus, paired t-tests for each age group were calculated.

Results – First Fixation: Over the three test trials and age groups, out of 543 possible anticipations, participants showed anticipations towards the interrupted path in 225 trials (41.44%). The 2-year-olds anticipated to the interrupted path in 74 out of 126 trials (58.73%). Of

the 74 trials, 2-year-olds anticipated towards the gap first in 13 trials (17.57 %). A Chi-Square-Test between the number of first anticipations towards the path and the gap turned out to be significant, with $\chi^2(1) = 31.14$, $p < .001$. The group of the 5-year-olds anticipated towards the interrupted path in 86 out of 141 trials (60.99%). Thereof they anticipated towards the gap first in 7 trials (8.14%); the Chi-Square-Test between the number of first anticipations towards the gap and the path was significant, with $\chi^2(1) = 60.28$, $p < .001$. Thus, results show that 2- and 5-year-olds looked first at the path and not at the gap. Results of young and older adults depict the same pattern. Younger adults anticipated towards the interrupted path in 34 of 135 trials (25.19%). From these trials they looked first at the gap in 1 trial (2.94%), the Chi-Square-Test turned out significant with $\chi^2(1) = 30.12$, $p < .001$. Older adults anticipated towards the interrupted path in 31 of 141 trials (21.99%), from which they looked in 3 trials at the gap first (9.68%). The Chi-Square-Test turned out significant with $\chi^2(1) = 20.16$, $p < .001$.

Furthermore, the individual test trials over all age groups were analyzed. Results showed that for the first test trial, 79 participants anticipated towards the interrupted path. Five of the 79 participants looked first towards the gap. The Chi-Square-Test showed a significant difference in frequencies with $\chi^2(1) = 60.27$, $p < .001$. In the second test trial, 80 participants anticipated towards the interrupted path; 10 of these 80 participants looked first towards the gap. With $\chi^2(1) = 45.00$, $p < .001$, the Chi-Square-Test revealed a significant difference. Similarly, 66 participants anticipated towards the interrupted path in the third test trial and 9 of the 66 participants fixated first the gap. Again, the Chi-Square-Test was significant with $\chi^2(1) = 34.91$, $p < .001$.

Results – Total Looking Time: Descriptives of the total looking time towards the gap and the path of the individual test trials for each age group can be found in Table 1. The paired t-tests for the 2-year-olds in all three test trials turned out significant, with $t(27) = -2.38$, $p = .025$ for

Trial 1, $t(28) = -2.05$, $p = .0499$ for Trial 2 and $t(17) = -3.22$, $p = .005$ for Trial 3. This indicates that overall, 2-year-olds looked longer towards the path than the gap in all three test trials. A similar pattern revealed the analysis of the 5-year-olds. The total looking time towards the path was significantly longer than towards the gap in Trial 1 with $t(31) = -8.16$, $p < .001$, in Trial 2 with $t(28) = -5.42$, $p < .001$, as well as in Trial 3 with $t(24) = -3.49$, $p = .002$. For the younger adults, all three paired t-tests were significant as well with $t(8) = -4.40$, $p = .002$ for Trial 1, $t(10) = -3.25$, $p = .009$ for Trial 2, and $t(13) = -3.50$, $p = .004$ for Trial 3. As can be seen in Table 1, they looked longer towards the path than the gap. A similar pattern revealed the analysis of older adults, with significant paired t-tests of Trial 1 with $t(10) = -4.28$, $p = .002$ and Trial 2 with $t(10) = -4.94$, $p = .001$. The comparison of Trial 3 was not significant, with $t(8) = -1.65$, $p = .138$.

Table 1.

Mean scores and standard deviations of the Total Looking Time towards the AOI of the gap and AOI of the path for each of the three test trials.

	**Trial 1**		**Trial 2**		**Trial 3**	
	**Gap**	**Path**	**Gap**	**Path**	**Gap**	**Path**
**2-year-olds**	0.57 (0.11)	1.05 (0.16)	0.6 (0.13)	0.87 (0.14)	0.3 (0.07)	0.93 (0.19)
**5-year-olds**	0.26 (0.07)	1.82 (0.16)	0.36 (0.07)	1.23 (0.14)	0.27 (0.08)	1 (0.18)
**Younger adults**	0.2 (0.14)	1.29 (0.26)	0.34 (0.14)	1.93 (0.41)	0.18 (0.11)	0.94 (0.15)
**Older adults**	0.15 (0.08)	0.71 (0.1)	0.14 (0.07)	0.66 (0.09)	0.23 (0.12)	0.57 (0.18)
